# Genetic and Environmental Influences on Sweet Taste Liking and Related Traits: New Insights from Twin Cohorts

**DOI:** 10.1007/s10519-025-10232-2

**Published:** 2025-09-19

**Authors:** Rhiannon M. Armitage, Vasiliki Iatridi, Darya Gaysina, Hely Tuorila, Martin R. Yeomans, Jaakko Kaprio, Stephanie Zellers

**Affiliations:** 1 School of Psychology, University of Sussex, Brighton, BN1 9QH UK; 2 Department of Sport, Health Sciences and Social Work, Oxford Brookes University, Oxford, UK; 3 Department of Food and Nutrition, University of Helsinki, Helsinki, Finland; 4 Department of Consumer and Food Sciences, University of Pretoria, Pretoria, South Africa; 5 Institute for Molecular Medicine Finland FIMM, University of Helsinki, Helsinki, Finland

**Keywords:** Sweet-liking, Heritability, Twin-modelling, Adults, Humans, European ancestry

## Abstract

Reducing sugar intake is a key component of global health policies and dietary guidelines. However, individuals vary substantially in sweet-liking, commonly characterized by sweet-liking status (extreme sweet-likers, moderate sweet-likers, and sweet-dislikers), yet the heritability of these categories remains unexplored. Monozygotic and dizygotic twins from Finland (FinnTwin12; *n* = 468; 60% female, aged 21–24) and the UK (TwinsUK; *n* = 967; 90% female, aged 18–81) rated their liking and perceived intensity of a 20% (w/v) sucrose solution, reported their liking and consumption-frequency of food and beverages and completed additional behavioral, eating and personality measures. We estimated the contribution of additive genetic (A), nonadditive genetic (D), shared (C), and unshared environmental factors (E) in the variance and covariance of sweet-liking (defined ordinally through sweet-liking status and continuously) with related traits to see if they share similar proportions of genetic and environmental factors. Model-fitting indicated 30–48% of the variability in sweet-liking was attributed to (A) additive genetic factors and 52–70% to (E) environmental exposures not shared by siblings. Importantly, such AE models consistently fit best, regardless of sex, cohort, or sweet-liking assessment method. Broadly, correlations between sweet-liking and behavioral, eating, and personality measures were modest (–0.19 to 0.21), mostly positive and largely driven by shared genetic rather than environmental factors, with the strongest relationship seen for reported liking, consumption-frequency and craving for sweet foods. We demonstrate that unshared environment modulates individual differences in sweet-liking alongside a substantial genetic component that is partly shared with reported liking, consumption-frequency and craving for sweet foods.

**Supplementary Information:**

The online version contains supplementary material available at 10.1007/s10519-025-10232-2.

## Introduction

Sugar intake is a key focus in government policies (e.g., Backholer et al. 2017; Colchero et al. 2016; Luiten et al. 2016) and dietary guidelines globally (reviewed in Herforth et al. 2019; Willett et al. 2019) to address worldwide health concerns, including dental caries, type-2 diabetes and the obesity epidemic. In the UK, fewer than 1 in 10 children (9%) and 1 in 5 adults (19%) met the current recommendation for free sugar intake (Roberts et al. 2025). While this is less clear globally due to methodological and reporting differences, the intake of added and free sugars consistently exceeds dietary guidelines, particularly among school-aged children and adolescents (Walton et al. 2023).

From an evolutionary perspective, it is believed that taste systems initially evolved to inform us about the nutritional value or toxicity of food stimuli to promote biological fitness (Drewnowski et al. 2012). Most mammalian neonates, including humans, have an innate predisposition to prefer sweetness, as evidenced by positive facial expressions and sucking responses to sweet tastes in infancy (Desor et al. 1973; Maone et al. 1990; Rosenstein and Oster 1988; Steiner et al. 2001) and the womb (Liley 1972; Ventura and Worobey 2013). This innate liking for sweet taste is generally believed to predispose children to be attracted to breastmilk and fruits and support heightened energy requirements for growth (Coldwell et al. 2009; Mennella et al. 2014), as evolutionarily, sugar serves as a quick, safe and nutritious energy source (Drewnowski et al. 2012). However, while exposure to sweet tastes and broader perceptual, physiological and cognitive factors likely contribute to our preferences (discussed in Coldwell et al. 2009), even children can show disliking responses, questioning the universality of sweet liking (Bobowski and Mennella 2017; Reed et al. 2006).

**Fig. 1:**
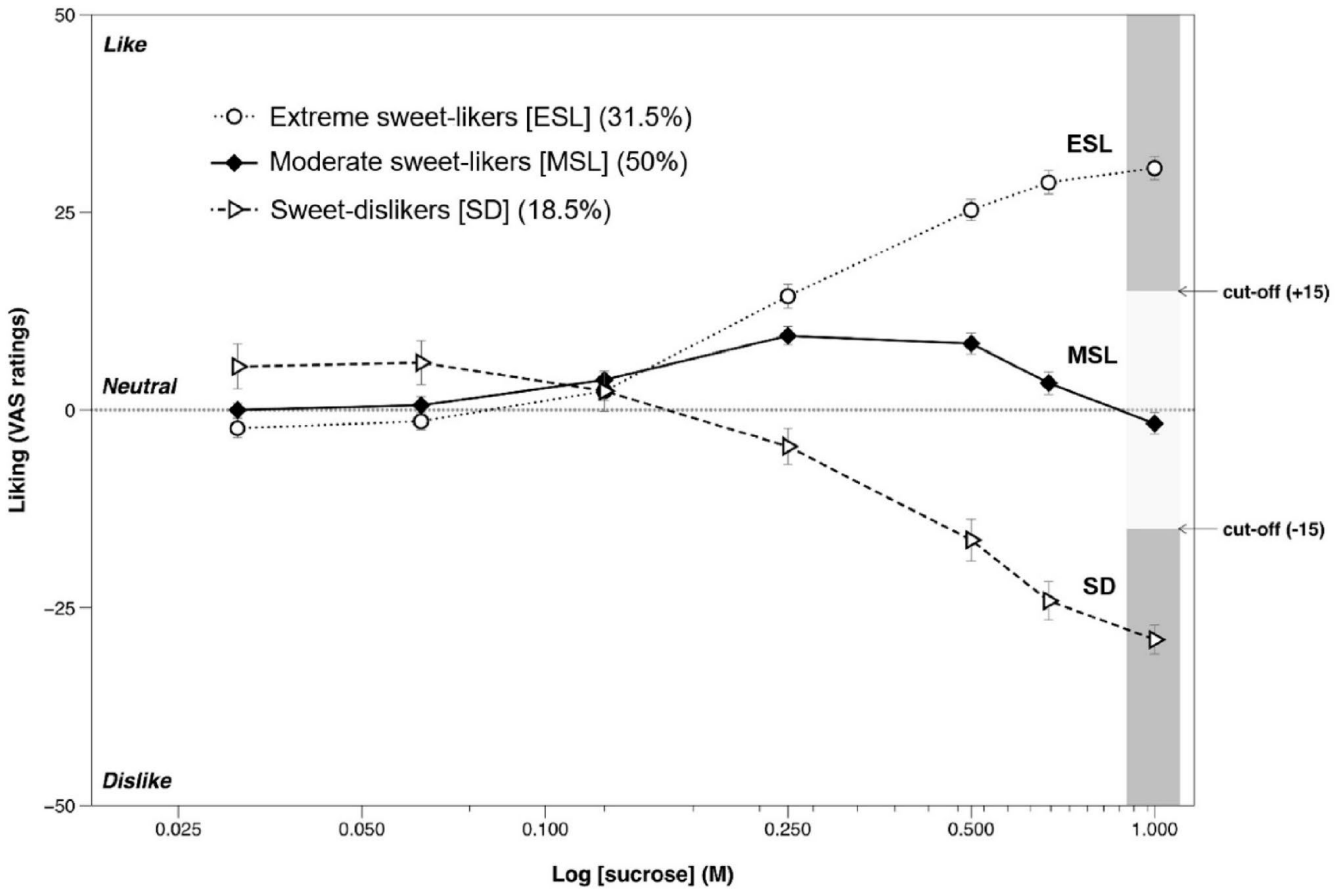
Liking response patterns to different sucrose intensities for the three sweet-liking status groups, as defined by hierarchal cluster analysis (modified with permission from (Iatridi et al. 2019): extreme sweet-likers (ESL), whose liking increased with sweetness intensity; moderate sweet-likers (MSL), whose liking peaked at 0.25 M sucrose before decreasing at the higher intensities extreme sweet-likers enjoy; and sweet-dislikers (SD), whose liking decreased with sweetness intensity. See (Iatridi et al. 2019) for analysis of the sensitivity and specificity scores to decide group classification cut-offs dependent on sucrose concentration.

Supporting this idea, over half a decade of research, sparked by the seminal work of Pangborn (Pangborn 1970), has established individual differences in liking for sweet tastes, with a recent wave of more statistically robust studies across populations in Europe, North America and Asia (e.g., Garneau et al. 2018; Iatridi et al. 2019; Kavaliauskaite et al. 2023; Kim et al. 2014; Lim et al. 2020; Yang et al. 2019) demonstrating a three group response pattern: extreme sweet-likers whose liking increases with sweetness intensity; moderate sweet-likers who show a mild liking for moderate levels of sweetness, which decreases at the high sweetness intensity extreme sweet-likers enjoy (i.e., an inverted-U shape response), and sweet-dislikers who show increasing dislike as sweetness increases (see Fig. 1). Our recent review highlighted the state of knowledge about sweet-liking status and their associations with body size and composition, dietary intake and behavioral measures (Armitage et al. 2021). Although distinct sweet-liking patterns exist across cultures, ethnicities and age groups (Ventura and Mennella 2011), the underlying reasons for these differences remain largely unknown and underexplored.

Similar to many complex behavioral traits, human liking for sweet tastes is likely an interaction between the body’s biological mechanisms, environmental exposures and experiences, as well as how we process and ultimately adapt to these biological and environmental signals over time (Mennella et al. 2016). However, given the drastic changes in the availability of sweetened products in modern food environments, most of the world no longer faces the same risks of food scarcity and uncertainty. As a result, this enhanced predisposition to like sweet tastes may not be as adaptative, and further variations in the preferred level of sweetness are expected. Environmental exposures and experiences play an essential role in facilitating eating preferences, likes and dislikes (e.g., Sullivan and Birch 1990). For instance, disliking can emerge through conditioned learning, such as the learned association between the taste of a food and an illness (e.g., Chambers 2018) or the cognitive evaluation of the consequences of specific food choices (e.g., Bernstein and Webster 1980; Hayes 2020). On the other hand, our underlying genetics may predispose us to behave and react more or less adaptively to the consequences of particular environments, such as being more susceptible to external cues of modern obesogenic environments (Llewellyn et al. 2023). Likewise, while exploring potential explanations for the different sweet-liking statuses, we proposed that the consistent proportions of these groupings may indicate a genetic basis, with a smaller proportion of sweet-dislikers and extreme sweet-likers compared to moderate sweet-likers (Armitage et al. 2021). Furthermore, the sweet-disliker group is evident across all ages and in people with differing ancestries (Armitage et al. 2021), suggesting a fundamental difference between individuals that may be rooted in genetics, at least in part.

Several studies have investigated the genetic underpinnings of sweet liking as a continuous trait, predominantly focusing on sweet-food liking. For example, genome-wide association studies (e.g., Fernández-Carrión et al. 2022; Hwang et al. 2019; Kawafune et al. 2020), candidate genes (reviewed in Bachmanov et al. 2011; Diószegi et al. 2019), and twin modelling studies (e.g., Pallister et al. 2015; Smith et al. 2017; Treur et al. 2016) have employed food preference or liking questionnaires to investigate sweet-food liking. Notably, three studies have directly explored the genetic factors associated with sweet taste liking using sucrose solutions (Keskitalo et al. 2007a, b; Keskitalo, Tuorila, Keskitalo et al. 2007a, b; Knaapila et al. 2012). Keskitalo and colleagues (Keskitalo et al. 2007a, b; Keskitalo, Tuorila, Keskitalo et al. 2007a, b) conducted two related studies utilizing linkage analysis in Finnish families (*n* = 146) and twin modelling in female British twins (*n* = 663). Specifically, genetic factors accounted for approximately 41–49% of the variation in liking for 18.75% and 20% (w/v) sucrose solution. In comparison, Knaapila and colleagues (Knaapila et al. 2012) explored the genetic influences of broader chemosensory traits using twin modelling and a candidate gene study, including liking ratings for a 12% (w/v) sucrose solution in an American sample (*n* = 572: 80% female). They found that 27% of the variability was due to genetic factors in the less intense sucrose solution (0.35 M vs. 0.55/0.58 M) and, interestingly, noted a distinct reduction in sweet-liking as participants aged.

Critically, however, most of those studies did not take into account individual variability in liking patterns for sweet taste (i.e., sweet-liking is not linear, constant across sweetness intensities or universal), and none of those studies grouped participants based on their hedonic reactions to sweet taste using a standardized protocol (referred to as “sweet-liking status” in this paper but also often called “sweet-liking phenotyping” in sensory research). Therefore, here we build on previous literature to explore for the first time the heritability of sweet-liking status through twin modelling to see if the variation in sweet-liking is due to genetic factors which add together to affect a trait (A: additive genetic), interact to create unique traits (D: nonadditive genetic) alongside environmental factors which are common and make individuals within families more similar (C: shared environment) or unique and make individuals differ (E: unshared environment). To facilitate comparisons with previous research, identify the best fit of the data for explaining individual differences in sweet liking, and assess the impact of operational definitions on quantitative genetic estimates, we define sweet-liking both ordinally through sweet-liking status and as a continuous variable.

We then examine whether correlations between sweet-liking assessment methods and related traits (i.e., sweet food liking, dietary intake, personality and eating behavior measures) share similar proportions of genetic or environmental factors in a genetically informative sample of identical (monozygotic) and non-identical (dizygotic) twins using two distinct populations from the UK and Finland. There are limited twin datasets with chemosensory data; therefore, we use an expanded version from Keskitalo and colleagues (Keskitalo et al. 2007) (*N* = 1435 vs. 663), which includes a Finnish cohort alongside both male and female twins. Furthermore, we incorporate a comprehensive range of related traits not previously investigated, improved statistical methods to reduce type-1 errors and parameter bias (i.e., direct symmetric model parameterization: Verhulst et al. 2019), and to avoid conflating sweetness or carbohydrates with fat, to ensure liking for sweet and sweet-fat foods is distinctly separated, we categorize food and beverages based on their sensory, macronutrient, and sensory-macronutrient properties.

## Method

### Participants

The present data were collected concurrently in British and Finnish twin research units as part of a wider project between 2005 and 2007. The British participants (*n* = 967 twin individuals) were recruited from the UK Adult Twin Registry (Spector and Williams 2006) and approved by the Guy’s and St Thomas’s Hospital Ethics Committee. Testing of the Finnish participants (*n* = 468 twin individuals) was part of the fourth wave assessment of the FinnTwin12 study (Kaprio et al. 2002), based on five consecutive and complete year cohorts of Finnish twins born in 1983–87. **Table 1** presents participants’ demographics by cohort. The Coordinating Ethics Committee of Helsinki University Hospital and the IRB of Indiana University, Bloomington, Indiana, U.S.A approved this research. The data analysis in the present study was approved by the University of Sussex Sciences & Technology Cross-Schools Research Ethics Committee (protocol ER/RA294/11) and conducted per the ethical standards laid down by the British Psychological Society and the Declaration of Helsinki.

### Measures

Both cohorts followed the same protocols, with data collected in English (TwinsUK) or in Finnish (FinnTwin12). Each twin was sent a postal questionnaire with a range of measures tracking behaviors, personality traits and attitudes completed at home and brought to a 3-6-hour clinic visit, which included several clinical tests and a taste test (Tuorila et al. 2017). Zygosity was determined using the “Peas in the Pod” questionnaire (Martin and Martin 1975) in the UK and “Kuten kaksi marjaa” questionnaire in Finland (Sarna et al. 1978), and confirmed by genotyping.

### Assessing sweet-taste Liking

A single solution of 20% (w/v) sucrose (0.58 M) was selected based on previous piloting to establish the heritability of suprathreshold sucrose liking, taking into account ease and time to prepare and administer (Keskitalo et al. 2007a, b; Keskitalo, Tuorila, Keskitalo et al. 2007a, b; Laing et al. 1993). Solutions were prepared the night before testing by dissolving a 4 g pre-packaged sugar sachet (Finnsugar, Kantvik, Finland) into a standard-size plastic cup, marked at 4 cL and 2 cL, filled with water to the 2 cl line (Polarcup, Hämeenlinna, Finland). Solutions were stored in the refrigerator at 7 ◦C and brought to room temperature before testing.

Participants attended the clinic after overnight fasting. They were given oral and written instructions for the taste test and were instructed not to communicate with other participants if they were present. However, the test administrator was present throughout. First, participants received instructions to rinse their mouths with water, after which they emptied the entire 20-ml sweet solution into their mouth and swilled for 5–10 s before expectorating. Immediately after, they rated the solution for liking using a 120-mm vertical Labeled Affective Magnitude scale (LAM: Schutz and Cardello 2001) end anchored with 0, “the greatest imaginable disliking,” and 120, “the greatest imaginable liking”, and intensity using a 120-mm vertical Labeled Magnitude Scale (LMS: Green et al. 1993), ranging from 0, “No sensation” to 120, “The strongest sensation imaginable”. After this, participants rated the intensity of a 6-n-propylthiouracil (PROP) filter paper (20) as a positive control for heritability (Keskitalo et al. 2007) following procedures described in (Keskitalo et al. 2007a, b).

### Food Liking and Intake

Participants provided ratings for their degree of liking and frequency of consumption for 34 (British) or 38 (Finnish) sweet or non-sweet foods and eight sweet or non-sweet beverages. These ratings used a 7-point hedonic scale from 1, “dislike very much”, to 7, “like very much”, for British participants or 1, “very unpleasant” to 7, “very pleasant” for Finnish participants, as the Finnish language lacks the word dislike. In both scales, 4 represents a neutral rating. For comparison and use of scales anchored by pleasantness and liking, see (Tuorila et al. 2008).

We assessed habitual intake of the same foods and beverages using corresponding food frequency questionnaires, where frequency of consumption ranged from 1, “never”, to 6, “several times a day” (Keskitalo et al. 2007a, b). Subsequently, we grouped individual food and beverage items and calculated the mean consumption, considering their sensory (Keskitalo et al. 2008), macronutrient (Geiselman et al. 1998), and sensory-macronutrient (Hill et al. 1987) profiles (**Table **S1). We adapted the sweet grouping from Keskitalo et al. (Keskitalo et al. 2008), as sweet dislikers are known to tolerate sweet taste when combined with fat, and their grouping contained sweet-fat items.

### Health and Taste Attitude Scales

Both cohorts completed two subscales of the Health and Taste Attitude Scales (Roininen et al. 2001). Participants rated six statements quantifying Craving for Sweet Foods, such as “I often have cravings for sweets”, and eight statements quantifying the General Health Interest, including “I am very particular about the healthiness of food I eat” on a scale of 1, “strongly disagree” to 7, “strongly agree”. The final instrument scores ranged from 1 to 7.

### Revised Version of the Three-Factor Eating Questionnaire

The revised version of the Three-Factor Eating Questionnaire (TFEQ-R18; Karlsson et al. 2000) was completed by both cohorts, measuring cognitive restraint (6 items including “How likely are you to consciously eat less than you want?”: theoretical range 6–24), uncontrolled eating (9 items including “How often do you feel hungry?”: theoretical range 9–36), and emotional eating (3 items including “When I feel anxious, I find myself eating”: theoretical range 3–12).

### Food Neophobia

The Food Neophobia Scale (FNS; Pliner and Hobden 1992) consists of ten statements, such as “I don’t like new foods,” rated from 1, “strongly disagree” to 7, “strongly agree”, with final scores ranging from 10 to 70.

### Personality Inventory

The Neo Five-Factor Inventory (NEO-FFI) is derived from the larger item pool of the NEO Personality Inventory (John and Srivastava 1999) and adapted to shorter 60-items in English (TwinsUK: Costa and McCrae 1985) and 67-items in Finnish (Paunonen et al. 2003; Pulver et al. 1995). The NEO inventories measure five major personality dimensions from 0, “strongly disagree” to 4, “strongly agree”: Neuroticism (vs. emotional stability), Extraversion (vs. introversion), Openness (vs. closedness to experience), Conscientiousness (vs. lack of direction), and Agreeableness (vs. antagonism) (John and Srivastava 1999). The score for each dimension is the mean of responses for all items in that dimension.

### Analytical Strategy

The research questions, hypotheses and analytical strategy were pre-registered on Open Science Framework prior to data access (https://osf.io/rzkqu). All analyses were run using OpenMx version 2.21.11 (Neale et al. 2016) in *R* (Version 4.4: R CoreTeam 2023).

### Determining sweet-liking Status

We determined sweet-liking status using ratings for a 20% (w/v) sucrose solution (0.58 M) and either treated sweet-liking as a continuous variable after scale transformations or categorized participants following a sensitivity specificity analysis (Iatridi et al. 2019), which determined the optimal cut-off for sweet-liking status characterization. This categorization has been used in multiple follow-up studies and larger trials (e.g., Boxall et al. 2025; Čad et al. 2025; Cheung et al. 2024). Here, liking ratings were first standardized by translating ratings to a 100-point scale (-50 to + 50). Following this, we used the liking cut-off score with the highest combined sensitivity and specificity for the closest solution specified by Iatridi et al. (2019) (0.5 M), which suggested for the classification of an extreme sweet-liker, liking ratings had to be higher than + 15, for sweet-dislikers below 0, and ratings between 0 and + 15 for a moderate sweet-liker.

### Intraclass Correlations

To support the multivariate twin analysis, we first calculated the mean ratings, standard deviations, and within-pair intraclass correlations using Pearson’s *r* correlation coefficient for the personality, behavioral and eating traits of interest for both monozygotic and dizygotic twins separately, exploring the effects of age, sex, and cohort. If within-pair correlations were higher for monozygotic twins than dizygotic twins, this would suggest that genetic effects contribute to the traits (Rijsdijk and Sham 2002). Depending on the difference between the strength of these correlations for the monozygotic twins compared to dizygotic twins, this could also suggest which quantitative twin model may be most appropriate as a starting point: (1) additive genetic, shared environmental, and unique environmental effects (ACE model) where the difference is less than twice as strong in monozygotic twins, or (2) additive genetic, genetic dominance, and unique environmental effects (ADE model) where the correlation is over twice as strong. In addition, we also explored Pearson’s correlation coefficients between the traits of interest. Significant correlations could suggest that there are common factors underlying these traits.

### Quantitative Genetic Analysis

Classic twin modelling, which partitions the phenotypic variance of a trait, relies on several assumptions. Monozygotic twins are genetically identical at the sequence level, whereas dizygotic twins share, on average, half of their segregating genes. We assume that relevant environmental exposures do not differ significantly between monozygotic and dizygotic twin pairs and that genetic and environmental influences are independent and additive. Therefore, there are no modelled influences on the population variance due to gene-environment correlation (rGE), gene-environment interaction (GxE), sex limitation, gene-age interaction, or epistasis.

In genetic modelling, we treat these variance components as latent (unmeasured) and standardized independent variables to explain the variation of the trait of interest (our dependent variable), in this case, sweet-liking. We calculate the variance components explaining the total observed phenotypic variance by squaring the model’s path coefficients (regression coefficients). For the genetic components, the correlations of both additive and nonadditive genetic effects are 1 within monozygotic pairs. However, the correlations are 0.5 for additive and 0.25 for nonadditive genetic effects within dizygotic pairs.

Based on these assumptions, the phenotypic variance of a trait can be decomposed into additive genetic effects (A), dominant (nonadditive) genetic effects (D), shared (common) environmental effects (C), and unshared environmental effects (E). Due to the structure of the data only containing twins reared together, without other relatives or adopted twins, we were only able to estimate shared environment variance (ACE model) or dominant genetic variance (ADE model) separately and then compare the models and their submodels to see which combination of the variance components described above best explained the data.

The twin model also relies on certain assumptions about the data: the mean and variance of the trait of interest do not differ systematically between twins within a pair or between monozygotic and dizygotic twins. We tested these assumptions by first fitting a saturated model, where phenotype means and variances are freely estimated separately for each twin within a pair and by zygosity. We then compared this saturated model to increasingly restrictive models corresponding to each assumption, with comparisons via likelihood ratio tests. A significant *p*-value at the alpha level of 0.05 suggested a violation of that assumption.

### Univariate Twin Models

Univariate twin models decompose the variation in one phenotype into either ACE or ADE sources and their respective submodels. We compare full models to each other (ACE and ADE) and submodels (ex. ACE to AE, CE, and E; ADE to AE, DE, and E) to the full model via likelihood ratio tests. A significant *p*-value indicates retaining the variance component, whereas a non-significant *p*-value suggests removing a variance component from the model.

To avoid the upward biasing of parameter estimates in Cholesky decomposition models, we used a direct symmetric model parameterization (Verhulst et al. 2019) to estimate univariate models for sweet-liking. We operationalized sweet-liking both as a continuous and ordinal variable, as a sensitivity analysis for how researcher choice may impact the quantitative genetic results. Continuous twin models directly decompose the variation in the observed phenotype, whereas ordinal twin models assume that the observed categories reflect an underlying latent liability with various thresholds. We can interpret these thresholds as z-scores by fixing the mean and variance of the latent liability at 0 and 1, respectively. We compared results between the ordinal and continuous models by evaluating confidence intervals for comparable parameters and parsimony.

We included age centered at the sample mean as a covariate for all quantitative genetic models and stratified univariate models of sweet liking by cohort (British or Finnish) and sex. Additionally, we included Finnish opposite-sex dizygotic twins. There were no British opposite-sex dizygotic twins.

We followed up univariate models with an evaluation of sex and cohort differences in the variance components underlying sweet-liking as there are known sex and age differences in sweet-liking, and the ages between cohorts varied greatly. We estimated and compared separate models for each sex and cohort where parameter estimates were constrained to equality or freely estimated via a likelihood-ratio test. A significant *p*-value indicates that the parameter differs between sexes or cohorts, whereas a non-significant *p*-value suggests that the parameter does not differ between sexes or cohorts. We first conduct an omnibus test, where all parameters are simultaneously constrained to equality between cohorts or sexes. If the omnibus test is significant, that would indicate the presence of some cohort or sex differences. We then follow up by testing each parameter individually.

### Bivariate Twin Models

We first evaluated individual-level Pearson correlations between sweet-liking rating and all other continuous measurements described in “Measures”: liking and consumption-frequency of foods grouped by sensory, macronutrient, and sensory-macronutrient profiles, BMI, PROP intensity, eating behaviors and health interests (i.e., food neophobia, general health interest, craving for sweet foods, cognitive restraint, uncontrolled and emotional eating), and personality (i.e., NEO-FFI: neuroticism, extraversion, openness, conscientiousness, and agreeableness). We then evaluated bivariate twin models for all variables significantly correlated with sweet-liking ratings to decompose the observed correlation into genetic and environmental components.

## Results

**Table 1** presents participants’ demographics by cohort. See **Table ****S2** for demographics by sweet-liking status. Overall, both cohorts were majority female, though the percent female was higher in the British cohort, which also had a greater age and BMI range than the Finnish cohort. The distribution of sweet-liking status also differed, with the highest proportion of extreme sweet-likers in the younger Finnish cohort, who preferred sweet taste more overall, and sweet-dislikers in the older British cohort.

**Table 1:** Summary of demographic and anthropometric characteristics.

	Finnish Twins (*n* = 468)		British Twins (*n* = 967)
Zygosity (Proportion, N)			
Monozygotic	45%, 209		49%, 476
Dizygotic Same Sex	32%, 148		51%, 491
Dizygotic Opposite Sex	24%, 111		-
Twin Pairs (Total N Pairs, % Complete Pairs)			
Monozygotic	111, 88%		244, 93%
Dizygotic Same Sex	80, 85%		250, 96%
Dizygotic Opposite Sex	68, 63%		-
Sex (Proportion, N)			
Male	40%, 186		10%, 101
Female	60%, 282		90%, 866
Weight Group (Proportion, N)			
Underweight	4%, 20		1%, 11
Healthy weight	70%, 328		43%, 414
Overweight	24%, 112		51%, 493
Obesity	2%, 8		5%, 49
Age			
mean ± s.d.	22.67 ± 0.47		55.64 ± 12.51
(range)	(21.25–24.54)		(18.23–80.71)
BMI			
mean ± s.d.	23.45 ± 3.88		26.33 ± 4.71
(range)	(17.21–42.91)		(16.9-48.57)
Liking Rating			
mean ± s.d.	60.29 ± 14.8		52.62 ± 19.7
(range)	13.33–95.83		0–95
Intensity Rating			
mean ± s.d.	31.21 ± 14.84		27.91 ± 18.3
(range)	(1.67–90.8)		0-100
Sweet-liking Status (Proportion, N)			
Extreme sweet-likers	44%, 206	36%, 346	
Moderate sweet-likers	32%, 148	22%, 217	
Sweet-dislikers	24%, 114	42%, 404	

Abbreviations: s.d., standard deviation

### Intraclass Correlations

As expected for genetically influenced traits, monozygotic twins were more strongly correlated than dizygotic twins; this was true for Finnish and British participants, and if sweet-liking was treated continuously or ordinally through sweet-liking status (**Table 2**).

**Table 2:** Summary of correlations for Finnish and British twins

	Finnish MZ	Finnish DZ	British MZ	British DZ
Continuous sweet-liking	0.34 [0.15, 0.51]	0.08 [-0.11, 0.26]	0.50 [0.40, 0.59]	0.21 [0.09, 0.33]
Ordinal Sweet-liking status	0.40 [0.11]	0.01 [0.12]	0.44 [0.07]	0.19 [0.08]

Note: For continuous rating, the correlation is a Pearson’s r, and the brackets represent 95% confidence intervals around the point estimate. For sweet-liking status, the correlation is a polychoric correlation, and the bracket represents the standard error for the point estimate

### Quantitative genetic analysis

All models met the three assumptions of the twin models as tested via comparisons of the saturated model to reduced models.

### Univariate Twin Models

We estimated univariate twin models to decompose the variation in sweet-liking into its components and evaluated the inclusion of each possible component (A, C, D, and/or E). For each sex group (British males, British females, Finnish males, Finnish females) within each cohort, a model containing only additive genetic and unique environmental components (i.e., AE model) fits the data best. We present ACE, ADE, AE, CE, DE, and E-only model comparisons in **Table ****S3**.

We followed up univariate models with an evaluation of sex and cohort differences in the variance components underlying sweet-taste liking (**Table S4**). No sex differences were identified in the continuous or ordinal models for the British or Finnish cohorts. However, we identified significant cohort differences in the continuous and ordinal models (**Table S4**) and further investigated these with tests of differences in individual parameters (**Table S5**). In the continuous model, the total variance of sweet liking was greater in the British cohort compared to the Finnish cohort. Mean sweet-liking was also significantly higher in the Finnish than the British cohort. In the ordinal models, significant cohort differences were found for the second threshold (i.e., the increment between moderate sweet-likers and sweet-dislikers) but not the first (i.e., extreme sweet-likers and moderate sweet-likers). Specifically, the British cohort had a larger proportion of sweet-dislikers (42%) than the Finnish cohort (24%).

We therefore present the best-fitting model and confidence intervals in **Table 3**, which is an AE model stratified by cohort but collapsed across sex. However, we include sex and age as covariates, as the literature suggests age (e.g., Knaapila et al. 2012; Ventura and Mennella 2011) and sex differences (e.g., Yang et al. 2020) in sweet-liking. Given the broad similarity in results between continuous and ordinal approaches, we present the remaining analyses using the continuous models and include the ordinal models in supplementary materials.

**Table 3:** Summary of best fitting models for Finnish and British twins

Parameter	Finnish Sample Estimate [95% CI]	British Sample Estimate [95% CI]
Continuous Model		
Heritability	0.30 [0.17, 0.43]	0.48 [0.39, 0.56]
Total Phenotypic Variance	218.5 [192.1, 249.8]	384.6 [350.5, 424.1]
**Ordinal Model**		
Heritability	0.33 [0.11, 0.53]	0.42 [0.28, 0.55]

### Pearsons Correlations

At the individual level, sweet-liking rating significantly correlated with several scales in Finnish and British cohorts. Heat maps depicting the individual correlations between all continuous variables are presented in **Figure ****S1****A and S1B**, and the associated *p*-values in **Table S6**. Broadly, phenotypic correlations between questionnaire scales and sweet liking were modest, ranging from − 0.19 to 0.21, and mostly positive (greater sweet liking associated with higher scale response). Overall, sweet-liking was significantly correlated with PROP intensity, sweetness intensity and the craving for sweet food scale in the British cohort and liking of various food groupings and, their consumption-frequency in both cohorts. Specifically, sweet-liking was correlated with the liking of 11 different food groupings and the consumption frequency of four food groupings in the Finnish cohort and the liking of 21 different food groupings, the consumption frequency of 15 food groupings. Sweet-liking was not significantly correlated with any other scale in either cohort (i.e., Food Neophobia Scale, the Three-Factor Eating Questionnaire, NEO Five Factor Personality Inventory, or the General Health Interest Scale).

### Bivariate Twin Models

We estimated the bivariate model between sweet-liking and all traits that significantly correlated at the individual level. As there were cohort differences but no identified sex differences, we ran bivariate models collapsed across sex but stratified by cohort. The correlated phenotypes were all moderately heritable, with heritability estimates ranging from 0.11 to 0.55. We decomposed the phenotypic correlations between sweet liking and questionnaire scales into additive genetic and unique environmental components. Genetic correlations were similarly modest but significant. However, for most traits, the unique environmental correlation did not significantly differ from 0, indicating that the phenotypic relationship between sweet liking and correlated traits may be due to shared genetic factors. A subset of 11 questionnaire scales predominantly relating to liking sweet food products (10/11), including three macronutrient, five sensory and two macronutrient-sensory groupings, was phenotypically correlated with sweet liking in both the British and Finnish cohorts; **Table 4** presents these results for continuous models, and **Table S7** contains the ordinal models. In these instances, the results agreed between the cohorts regarding the magnitude, direction, and decomposition of the correlation.

**Table 4:** Summary of best fitting bivariate results for Finnish and British twins using continuous model

Correlated Variable	Site	Heritability	Unique Environment	Phenotypic Correlation	Genetic Correlation	Unique Environmental Correlation
FLMacro_HFHCCHO: Food liking, macronutrients category of items high fat and high complex carbohydrates	Finland	0.40 [0.23, 0.54]	0.60 [0.46, 0.77]	0.19 [0.09, 0.29]	0.19 [-0.18, 0.52]	0.20 [0.02, 0.36]
	UK	0.40 [0.30, 0.49]	0.60 [0.51, 0.70]	0.12 [0.05, 0.18]	0.14 [-0.04, 0.32]	0.10 [-0.02, 0.21]
FLMacro_HFHS: Food liking, macronutrients category of items high fat and high simple sugars	Finland	0.52 [0.35, 0.65]	0.48 [0.35, 0.65]	0.20 [0.09, 0.30]	0.26 [-0.08, 0.57]	0.17 [-0.02, 0.35]
	UK	0.49 [0.40, 0.58]	0.51 [0.42, 0.60]	0.18 [0.12, 0.24]	0.36 [0.20, 0.52]	0.02 [-0.10, 0.13]
FLMacro_LFHS: Food liking, macronutrients category of items low in fat and high simple sugars	Finland	0.32 [0.14, 0.49]	0.68 [0.51, 0.86]	0.15 [0.04, 0.25]	0.09 [-0.33, 0.49]	0.18 [-0.01, 0.35]
	UK	0.43 [0.34, 0.52]	0.57 [0.48, 0.66]	0.16 [0.09, 0.22]	0.22 [0.05, 0.39]	0.11 [-0.01, 0.22]
FLSen_SweetFat: Food liking, sensory category of items sweet and fatty	Finland	0.53 [0.37, 0.65]	0.47 [0.35, 0.63]	0.20 [0.09, 0.30]	0.19 [-0.14, 0.50]	0.22 [0.03, 0.39]
	UK	0.50 [0.41, 0.59]	0.50 [0.41, 0.59]	0.18 [0.12, 0.25]	0.33 [0.17, 0.50]	0.04 [-0.08, 0.16]
FLSen_SweetFatExtra: Food liking, sensory category of sweet and fatty items with additional items	Finland	0.51 [0.35, 0.64]	0.49 [0.36, 0.65]	0.21 [0.10, 0.30]	0.20 [-0.13, 0.51]	0.22 [0.03, 0.38]
	UK	0.47 [0.37, 0.56]	0.53 [0.44, 0.63]	0.18 [0.12, 0.24]	0.34 [0.16, 0.51]	0.04 [-0.08, 0.16]
FLSenKes_SaltyFat: Food liking, sensory category of salty and fatty items from Keskitalo’s original groupings	Finland	0.36 [0.17, 0.52]	0.64 [0.48, 0.83]	0.15 [0.04, 0.25]	0.33 [-0.07, 0.71]	0.06 [-0.12, 0.24]
	UK	0.50 [0.41, 0.58]	0.50 [0.42, 0.59]	0.08 [0.02, 0.15]	0.17 [0.01, 0.33]	0 [-0.12, 0.12]
FLSenKes_Sweet: Food liking, sensory category of sweet items from Keskitalo’s original groupings	Finland	0.54 [0.37, 0.66]	0.46 [0.34, 0.63]	0.19 [0.09, 0.29]	0.14 [-0.18, 0.45]	0.24 [0.05, 0.41]
	UK	0.52 [0.43, 0.6]	0.48 [0.4, 0.57]	0.18 [0.12, 0.25]	0.31 [0.15, 0.46]	0.06 [-0.06, 0.18]
FLSenKes_SweetFat: Food liking, sensory category of sweet and fatty items from Keskitalo’s original groupings	Finland	0.53 [0.37, 0.65]	0.47 [0.35, 0.63]	0.20 [0.09, 0.3]	0.19 [-0.14, 0.50]	0.22 [0.03, 0.39]
	UK	0.50 [0.41, 0.59]	0.50 [0.41, 0.59]	0.18 [0.12, 0.25]	0.33 [0.17, 0.50]	0.04 [-0.08, 0.16]
FLSenMacro_HCSw: Food liking, sensory-macronutrient category of high carbohydrates and sweet items	Finland	0.46 [0.30, 0.59]	0.54 [0.41, 0.70]	0.13 [0.02, 0.23]	0.03 [-0.31, 0.36]	0.19 [0.01, 0.36]
	UK	0.44 [0.33, 0.53]	0.56 [0.47, 0.67]	0.11 [0.04, 0.17]	0.17 [-0.01, 0.32]	0.05 [-0.06, 0.17]
FLSenMacro_HFSw: Food liking, sensory-macronutrient category of high fat and sweet items	Finland	0.50 [0.33, 0.63]	0.50 [0.37, 0.67]	0.24 [0.13, 0.33]	0.26 [-0.08, 0.26]	0.23 [0.04, 0.39]
	UK	0.50 [0.40, 0.58]	0.50 [0.42, 0.60]	0.20 [0.13, 0.26]	0.33 [0.16, 0.42]	0.07 [-0.05, 0.19]
FUMacro_LFHS: Food use, macronutrients category of items low in fat and high simple sugars.	Finland	0.24 [0.07, 0.40]	0.76 [0.60, 0.93]	0.13 [0.02, 0.23]	0.20 [0.02, 0.71]	0.10 [-0.07, 0.27]
	UK	0.41 [0.30, 0.50]	0.59 [0.50, 0.70]	0.11 [0.05, 0.18]	0.08 [-0.09, 0.25]	0.14 [0.03, 0.26]

Note. Negative correlations here indicate that less sweet disliking (i.e., greater sweet liking) is associated with greater liking or use of various food types.

## Discussion

Despite the significant impact of taste hedonics on eating behavior and its implications for food preference, selection, and overall nutritional intake and health (reviewed in de Graaf and Boesveldt 2017), relatively few studies have investigated the determinants of individual differences in liking for sweet tastes. Of those that have, only three used sucrose solutions directly, and none have characterized sweet-liking by hedonic patterns commonly applied in sensory science (i.e., sweet-liking status), instead utilizing continuous metrics of sweet-liking. Therefore, to assess the impact of researchers’ choice on quantitative genetic results, we examined sweet-liking both as a continuous and an ordinal variable and its associations with related traits.

### Univariate models: genetic and environmental influences on sweet-liking across cohorts

Our univariate models examined potential genetic influences on sweet-liking, controlling for age and sex. Regardless of sex, cohort, or sweet-liking assessment method, an AE model consistently fit best. The heritability estimates were moderate, ranging from 30–48%, suggesting an innate genetic predisposition for sweet-liking that is also modifiable with a substantial unshared environmental influence (52–70%, including measurement error). These findings align with the three studies that directly explored the heritability of continuous sweet-taste liking using a real-life sweet taste: 27% heritability in the American, predominantly female sample (Knaapila et al. 2012), 41% in the Finnish family study (Keskitalo et al. 2007a, b) and 49% in the British, all-female cohort (Keskitalo, Tuorila, Keskitalo et al. 2007a, b), on which our sample builds. We found reasonable consistency in estimates within cohorts, with proportions between sweet-liking assessment methods varying 3–6%. Interestingly, as shown in Table 3, the discrepancies of heritability estimates between cohorts were smaller when using sweet-liking status (9% difference: British A = 42%; Finnish A = 33%; not statistically significant) than when treated as a continuous variable (18% difference: British A = 48%; Finnish A = 30%; statistically significant), further suggesting a potential benefit in classifying individuals by sweet-liking status (Tan and Tucker 2019).

Final models indicated no significant cohort differences, except for mean liking ratings, where the notably younger Finnish cohort liked sweet taste significantly more. This could reflect age-associated changes in sweet-liking, which generally follows a U-shaped liking pattern: peaking in childhood, decreasing with age until late adulthood, where it increases again (Venditti et al. 2020). Interestingly, this pattern was also noted by Knaapila et al. (2012). When we categorized participants by age group, sweet liking revealed a U-shaped response curve (**Table S9**); however, there are not enough twins in the same age categories between cohorts to directly compare. Differences between cohorts could also reflect cultural variations between individuals, differences in the ranges of sweet products available in each country (James 1990; Prättälä 2003), sex differences, or, most likely, a combination of factors (e.g., Venditti et al. 2020; Ventura and Mennella 2011; Yang et al. 2020). In contrast to past research, we did not see sex differences in sweet-liking. However, as our sample is predominantly female (67%), it may be underpowered to detect these (**Table S9**). We were also unable to evaluate age-related changes in heritability, due to the relatively small sample sizes, and this remains an important direction for future work.

We must also consider the complexity of eating behaviors, how these change over time, and the clear evidence presented here that sweet-liking is both heritable and shaped by the environment. Thus, it is reasonable that genetic influences on sweet liking may vary in magnitude across the lifespan, as previously reported for other behaviors (i.e., substance use: Zellers et al. 2022) and could also interact with the environment to influence eating behavior and related health concerns (Llewellyn et al. 2023) thereby predisposing an individual to respond differently to environmental factors. Furthermore, there may be critical periods in which genetics predisposes individuals to heightened sweet-liking. For example, the marked ‘innate’ liking for sweetness experienced during periods of high metabolic demands (i.e., childhood and adolescence: Coldwell et al. 2009; Mennella et al. 2014), which declines with age and environmental exposure to broader flavor profiles (e.g., Mennella et al. 2005).

### Bivariate models: genetic and environmental associations between related traits and sweet-liking

Our bivariate models explored the relatedness of wider traits not previously tested for their potential shared genetic relationship with sweet-liking that correlated in our initial descriptive correlations (i.e., liking and consumption-frequency of food items, craving for sweet foods, PROP intensity, sweetness-intensity). However, the correlations for most broader eating behaviors (i.e., food neophobia, cognitive restraint, uncontrolled and emotional eating) and personality traits measured by The Neo Five-Factor Inventory (i.e., neuroticism, extraversion, openness, conscientiousness, and agreeableness) were at most modest and not significant; therefore, they were not included in our final models. Our analyses revealed that most shared variance between sweet-liking and correlated measures was due to additive genetic factors. However, these shared genetic factors were modest in size (*r*_g_ range = − 0.20-0.50). The lack of significant environmental correlations may reflect low power to decompose the observed phenotypic correlations or other computational challenges related to confidence interval estimates.

The relationship between sweet-liking and PROP intensity was small (*r* = .07), however, this aligns with previous work that found while not significantly related, there was some association between sweet-liking status and PROP taster status (e.g., Yang et al. 2019; Yeomans et al. 2022). Sweetness intensity was negatively related to sweet-liking in the British cohort: the more a participant liked sweetness, the less intense their rating. This supports a broader body of literature showing negative experiences tend to be rated as more intense than those that are liked, although behaviorally, sweetness intensity seems to be rated similarly across the sweet-liking statuses (e.g., Armitage et al. 2021; Iatridi et al. 2019). The strongest shared relationship with sweet-liking classification was for liking ratings of various sensory and macronutrient groupings of sweet or sweet-fatty foods (*r* range = 0.16-0.22). However, we did not see the same strength of relationship for the consumption frequency of these same groupings. The phenotypic correlations were consistent across cohorts and the sweet-liking assessment method, suggesting a small shared genetic underpinning alongside substantial environmental influence that is not shared with sweet-liking.

Overall, these findings align with the broader literature on the distinct yet intertwined neurological pathways of ‘reward’ liking and ‘motivation’ wanting (Morales and Berridge 2020). Specifically, we found shared genetic relatedness between four overlapping but separate sweet-associated concepts that can conceptually be divided to associate more strongly with either ‘reward’ liking (i.e., liking for sweet tastes and sweet foods) or ‘motivational’ wanting (i.e., reported cravings for sweet products or the consumption-frequency of them). Notably, the strongest genetic correlation with sweet taste liking was liking for sweet foods, which may further suggest that the inherited underlying biology that drives phenotypic differences in sweet-liking could be rooted in enhanced reward processing in sweet-likers, who have also shown greater behavioral measures of reward sensitivity (e.g., Iatridi et al. 2020; Lippi et al. 2023). Although some shared genetic variance was found between liking for sweet taste and reported cravings for sweet products and their consumption-frequency, this was, to a lesser extent, supporting recent work from our group and others, which found that the sweet-liking statuses differed in liking for sweet products but not in consumption-frequency of them (Armitage et al. 2023; Čad et al. 2025). Interestingly, the largest component driving intake of sweet and sweet-fat foods was environmental. Thus, broader factors of food choice not related to sweet-liking may be better drivers of intake and protect against enhanced liking of sweet taste for extreme sweet-likers from translating into overconsumption.

### Strength, limitations, and future directions

This research is the first analysis to investigate the heritability of individual differences in sweet liking using sweet-liking status. Furthermore, it extends previous works by including female and male twins, a broader spectrum of potentially related traits and accounts for the sensory and macronutrient properties in dietary differences. While correlations between these related traits were small, given the complexities of eating behavior, these findings collectively contribute to a better understanding of how individual differences in sweet-liking fit with wider behavioral, eating and personality traits and take us one step closer to understanding how these may contribute to personalized health interventions (Spinelli and Monteleone 2021) and alternative public health strategies (Livingston 2018) designed to manage nutritional diseases.

However, it is essential to acknowledge the limitations inherent in secondary data analysis. Certain dimensions typically linked to sweet taste liking, such as broader anthropometric measures, reward processing, and interoception, were not examined. Likewise, although sweet liking was examined as a simple taste modality (sucrose solution) and in a real-life context (38-item food and 8-item beverage liking), only one sucrose solution was utilized (20% (w/v), which was also below the optimal concentration to distinguish between the sweet-liking statuses (Iatridi et al. 2019) and prevents replication of liking patterns. As such, this solution may have also been too weak to discriminate between sweet-liking status groups fully, and may partly explain why we did not observe the same distribution as seen in previous works using a higher concentration. The absence of more detailed dietary data beyond a survey (e.g., repeated dietary recalls, food diaries, real-life food-liking ratings), wider tastes (e.g., Prescott and Stevenson 2015) and sensory modalities (e.g., Piochi et al. 2021) may also limit calculations of disparities in dietary intake and real-life liking for wider sweet products. In addition, we recognize that our sample is from two affluent countries from European ancestry, so cannot be generalized to broader populations. Therefore, further research is needed to explore these relationships in wider ancestries that better reflect the breath of ethnicities, race, and cultures. Here we note a promising study exploring sweet-liking, using sweet-liking status, in individuals from underrepresented ancestry groups (Cheung et al. 2024).

We suggest future research should integrate more comprehensive dietary assessment, related measures (i.e., broader anthropometrics, reward processing and eating behaviors) and in-person taste tests into longitudinal twin study protocols in broader ancestry groups. Ideally, these would be conducted within broader epidemiology studies in a family setting (i.e., including genetic data, gut and metabolic markers from parents and family members), with whole genome data to allow gene mapping, genome-wide association studies and polygenic risk scores. This approach could elucidate the specific genetic and environmental factors that contribute to food intake, overconsumption and associated non-communicable diseases and track how these develop and change over time and with exposure to different environments (e.g., cultural, obesogenic). While the precise genes and environmental factors remain largely unknown, we can hypothesize key genes (discussed in Armitage et al. 2021), behavioral and environmental influences, which may act as protective or risk factors (e.g., availability, accessibility of foods, exposure, social norms, culture, socioeconomic status, health consciousness, personality traits, emotional eating, internal awareness of body signals etc.: Mozaffarian et al. 2018).

## Conclusions

We found an AE model with additive genetic and unshared environmental effects consistently fit best, regardless of sex, cohort, or sweet-liking assessment method. Compared to liking for sweetness as a continuous trait, sweet-liking status generated the most consistent estimates across cohorts, further supporting their use to classify and investigate individual differences in sweet taste liking. While unshared environment emerged as the larger source of variability in sweet-liking, additive genetics maintained a substantial role, notably co-varying with liking of specific sweet sensory and macronutrient food groupings, and to a lesser extent, their consumption-frequency and craving for sweet products, potentially supporting the distinction between liking and wanting mechanisms. A more holistic, interdisciplinary approach that can account for gene-environment interactions is needed in the future to elucidate the specific genes and environmental factors that contribute to individual differences in sweet-taste liking. Such work could offer crucial insights into the development of effective personalized approaches and alternative public health strategies aimed at managing nutritional diseases, including overconsumption, obesity, and related health concerns.

## Supplementary Information

Below is the link to the electronic supplementary material.

Supplementary Material 1

Supplementary Material 2

Supplementary Material 3

## Data Availability

Data described in the manuscript, code book, and analytic code will be made available upon request pending application, approval and payment to the Institute for Molecular Medicine Finland (FIMM), University of Helsinki, to access the FinnTwin12 cohort data and the Department of Twin Research & Genetic Epidemiology, King’s College London to access the TwinsUK data. For more information on the FinnTwin data, see https://www.helsinki.fi/fi/tutkimusryhmat/kaksostutkimus/yhteystiedot and for more information on TwinsUK see https://twinsuk.ac.uk/resources-for-researchers/our-data/.
