# Supplementary figures and images for: Genetic and Environmental Influences on Sweet Taste Liking and Related Traits: New Insights from Twin Cohorts
Source: Behav Genet. 2025 Sep 19;55(5):407–21. doi: 10.1007/s10519-025-10232-2 (PMC12494627; doi:10.1007/s10519-025-10232-2)

## FinnTwin Individual-Level Correlations

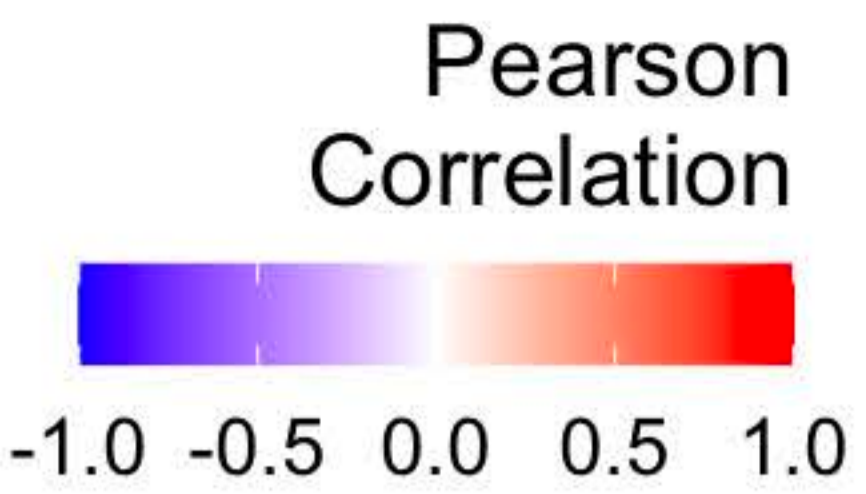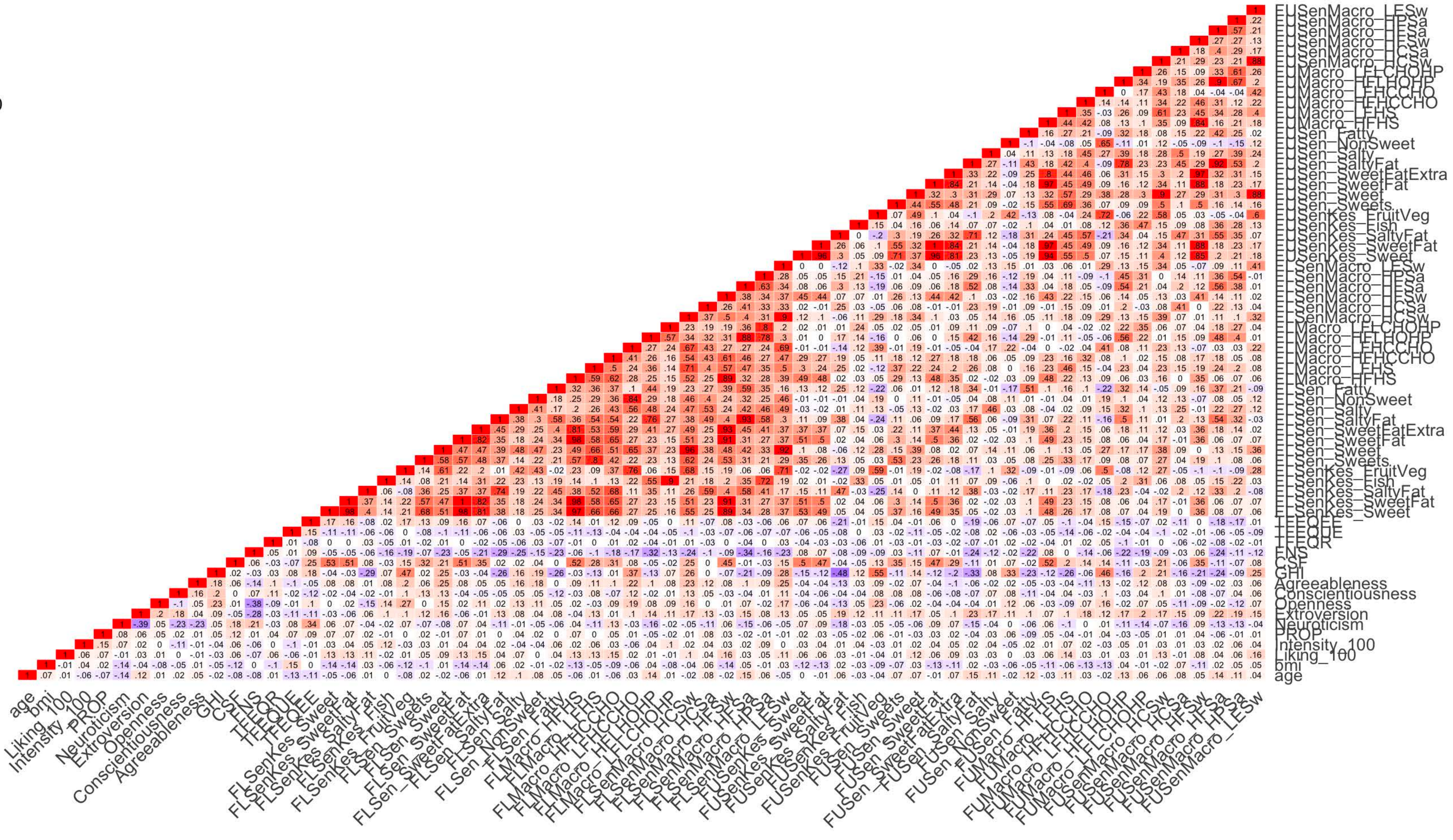

Supplement: Supplementary file 1 — Supplementary Material 1 [file 10519_2025_10232_MOESM1_ESM.pdf]

## TwinsUK Individual-Level Correlations

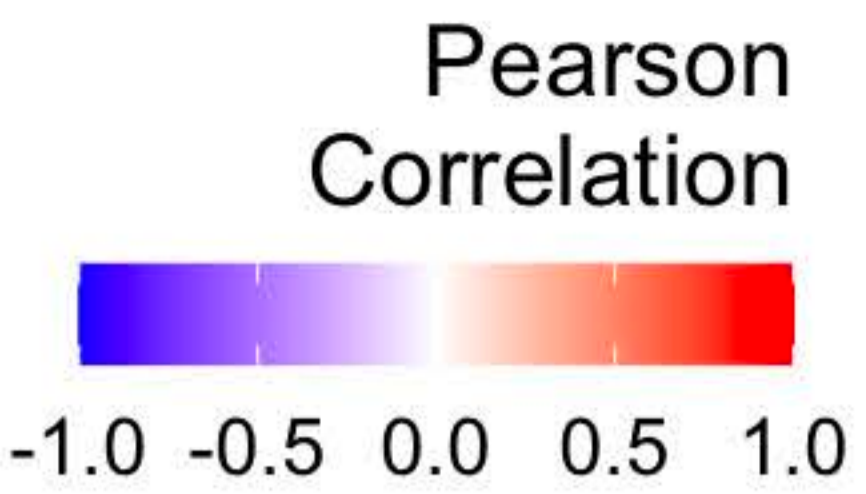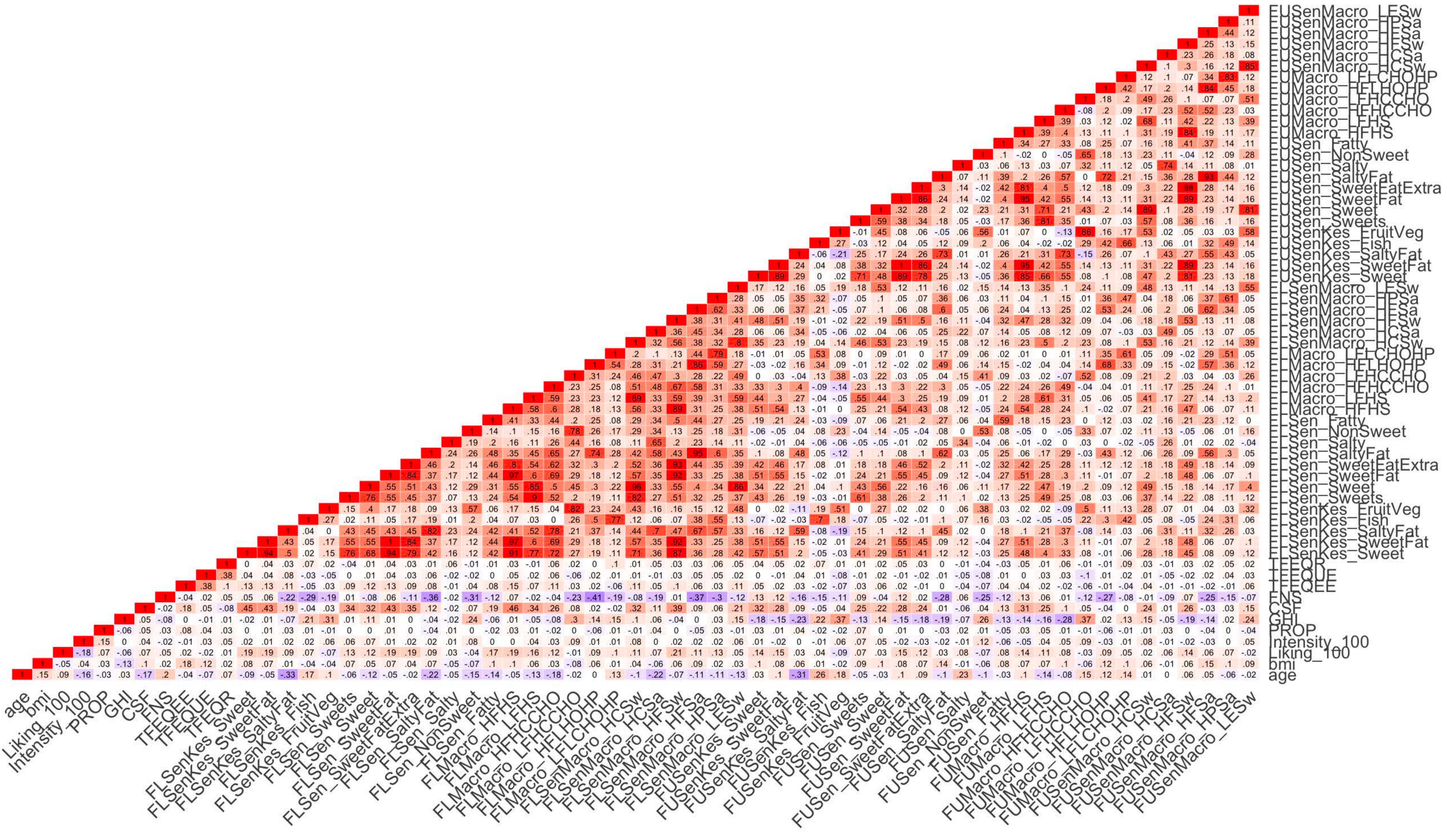

Supplement: Supplementary file 2 — Supplementary Material 2 [file 10519_2025_10232_MOESM2_ESM.pdf]
