# Supplementary material for: Genetic and Environmental Influences on Sweet Taste Liking and Related Traits: New Insights from Twin Cohorts
Source: Behav Genet. 2025 Sep 19;55(5):407–21. doi: 10.1007/s10519-025-10232-2 (PMC12494627; doi:10.1007/s10519-025-10232-2)
Supplement: Supplementary file 3 — Supplementary Material 3 [file 10519_2025_10232_MOESM3_ESM.pdf]

Supplemental Information: Genetic and environmental influences on sweet taste liking and related traits: new insights from twin cohorts

Rhiannon M. Armitage*[1], Vasiliki Iatridi[2], Darya Gaysina[1], Hely Tuorila[3,4], Martin R. Yeomans[1], Jaakko Kaprio[5] and Stephanie Zellers[5]

[1.] School of Psychology, University of Sussex, UK.

[2.] Department of Sport, Health Sciences and Social Work, Oxford Brookes University, UK.

[3.] Department of Food and Nutrition, University of Helsinki, Finland.

[4.] Department of Consumer and Food Sciences, University of Pretoria, South Africa.

[5.] Institute for Molecular Medicine Finland FIMM, University of Helsinki, Finland.

*Rhiannon M Armitage,

School of Psychology,

University of Sussex,

Brighton,

BN1 9QH,

UK

r.armitage@sussex.ac.uk

Table S1. Summary of liking and consumption-frequency food and beverage groupings by property

Food or Beverage Item	Included in sample		Food and Beverage Grouping		
	Finnish	British	Sensory*	Macronutrient**	Sensory-Macronutrient***
Bacon	No	Yes	FSa	HF/LCHO/HP	HF-Sa
Blue cheese	Yes	Yes	FSa	HF/LCHO/HP	HF-Sa
Chicken, turkey	Yes	Yes	U	LF/LCHO/HP	HP-Sa
Chocolate	Yes	Yes	SF	HF/HS	HF-S
Cold cuts	Yes	No	Sa	HF/LCHO/HP	HP-Sa
Cooked vegetables	Yes	Yes	S	LF/HCCHO	HC-S/LE-S
Cream products (e.g. sour cream, whipped cream)	Yes	Yes	F		
Drops	No	Yes	S	LF/HS	HC-S
Eggs	Yes	Yes	FU	HF/LCHO/HP	
Fish in batter	No	Yes	FSa		HF-Sa
Fresh vegetables	Yes	Yes	nS	LF/HCCHO	
Fried foods	Yes	Yes	FSa		HF-Sa
Fruit and berry juices	Yes	Yes	S	LF/HS	HC-S/LE-S
Hamburgers	Yes	Yes	FSa	n/a	HP-Sa
Ice-cream	Yes	Yes	SF	HF/HS	HF-S
Liquorice	Yes	Yes	S	LF/HS	HC-S
Meat	Yes	Yes	FU	HF/LCHO/HP	HF-Sa
Milk (Fullfat)	Yes	Yes	SF		HF-S
Milk (Semi-skimmed)	Yes	Yes	S		
Milk (Skimmed)	Yes	Yes	S		HC-S/LE-S
Mixed bread	Yes	No	nS	LF/HCCHO	
Other cheeses (e.g. Emmental, Brie)	Yes	Yes	FSa	HF/LCHO/HP	HF-Sa
Other fish	Yes	Yes	U	LF/LCHO/HP	HP-Sa
Pickled herring	Yes	Yes	SF		
Pizzas	Yes	Yes	FSa		HC-Sa

Potato (fried or French fries)	Yes	Yes	FSa	HF/HCCHO	HF-Sa
Potato (cooked or mashed)	Yes	Yes	Sa	LF/HCCHO	HC-Sa
Reduced-fat cheeses	Yes	Yes	FSa	HF/LCHO/HP	HF-Sa
Rice or pasta	Yes	Yes	nS	LF/HCCHO	
Rye bread	Yes	No	nS	LF/HCCHO	
Salami	Yes	No	FSa	HF/LCHO/HP	HF-Sa
Salmon or rainbow trout	Yes	Yes	FU	HF/LCHO/HP	
Salty paste	Yes	No	Sa		HF-Sa
Salty snacks	Yes	Yes	FSa	HF/HCCHO	HF-Sa
Sausage	Yes	Yes	FSa	HF/LCHO/HP	HF-Sa
Salad dressings	Yes	Yes	FSa		HF-Sa
Sweet desserts	Yes	Yes	SF	HF/HS	HF-S
Sweet fruits (e.g. pears, grapes, berries)	Yes	Yes	S	LF/HCCHO	HC-S/LE-S
Sweet pastry	Yes	Yes	SF	HF/HCCHO	HF-S
Sweet, low-calorie soft drinks	Yes	Yes	S		LE-S
Sweet, sugared soft drinks	Yes	Yes	S	LF/HS	HC-S/LE-S
Sweets	Yes	Yes	S	LF/HS	HC-S
Tuna	Yes	No	FU		HP-Sa
Vegetarian dishes	Yes	No	Sa	HF/HCCHO	
White bread	Yes	No	nS	LF/HCCHO	
Whole meats	Yes	No	U		HP-Sa

- Sensory categories: F = fatty; FSa = fatty-salty; nS = non-sweet; Sa = salty; S = sweet; SF = sweet-fatty
- Macronutrient categories: HF/HS = high fat and high simple sugars; HF/HCCHO = high fat and high complex carbohydrates; HF/LCHO/HP = high fat, low complex carbohydrates and high protein; LF/HS = low fat and high simple sugars; LF/HCCHO = low fat and high complex carbohydrates; LF/LCHO/HP = low fat, low complex carbohydrates and high protein
- Sensory-macronutrient categories: HC-S = high carbohydrates and sweet; HC-Sa = High carbohydrates and salty; HF-S = high fat and sweet; HF-Sa = high fat and high salty; HP-Sa = high protien and salty

Table S2. Summary of demographic and anthropometric characteristics by phenotype for Finnish and British twins

	Finnish Twins				British Twins			
	Extreme sweet-likers (n = 206) 44%	Moderate sweet-likers (n = 148) 32%	Sweet-dislikers (n = 114) 24%	Overall (n = 468)	Extreme sweet-likers (n = 346) 36%	Moderate sweet-likers (n = 217) 22%	Sweet-dislikers (n = 404) 42%	Overall (n = 967)
**Zygosity (%, n)**								
Monozygotic	44%, 90	45%, 66	46%, 53	45%, 209	50%, 174	51%, 111	47%, 191	49%, 476
Dyzygotic (Dz)	35%, 73	27.5%, 41	30%, 34	32%, 148	50%, 172	49%, 106	53%, 213	51%, 491
Dyzygotic (Osdz)	21%, 43	27.5%, 41	24%, 24	24%, 111	---	---	---	---
**Sex (%, n)**								
Male	45%, 92	34%, 51	38%, 43	40%, 186	13%, 44	12%, 25	8%, 32	10%, 101
Female	55%, 114	66%, 97	63%, 71	60%, 282	87%, 302	88%, 192	92%, 372	90%, 866
**BMI Category (%, n)**								
Underweight	6%, 12	3%, 4	4%, 4	4%, 20	1%, 3	1%, 2	1%, 6	1%, 11
Healthy weight	68%, 140	69%, 103	74%, 85	70%, 328	41%, 142	48%, 104	42%, 168	43%, 414
Overweight	25%, 52	25%, 37	20%, 23	24%, 112	53%, 185	48%, 104	50%, 204	51%, 493
Obesity	1%, 2	3%, 4	2%, 2	2%, 8	5%, 16	3%, 7	6%, 26	5%, 49
**Age (years)**								
mean±s.d.	22.69±0.46	22.63±0.42	21.68±0.53	22.67±0.47	56.78±12.84	55.82±12.77	54.57±12	55.64±12.51
(range)	(21.25-24.54)	(21.82-23.78)	(21.26-24.54)	(21.25-24.54)	(18.23-80.71)	(20.46-79.85)	(18.64-80.71)	(18.23-80.71)
**BMI**								
mean±s.d.	23.41±3.56	23.8±4.4	23.06±3.72	23.45±3.88	26.37±4.4	25.69±4.14	26.63±5.19	26.33±4.71
(range)	(17.21-36.83)	(17.68-42.91	(17.29-35.64)	(17.21-42.91)	(16.9-42.17)	(17.82-40.49)	(17.75-48.57)	(16.9-48.57)
**Liking**								
mean±s.d.	73.64±5.77	57.61±3.98	39.65±7.71	60.29±14.8	72.31±6.82	56.53±4.08	33.65±13.4	52.62±19.7
(range)	(65-95.83)	(50-64.14)	(13.33-49.17	13.33-95.83	65-95	50-64.17	0-49.17	0-95
**Intensity**								
mean±s.d.	33.31±16	27.86±12.51	31.77±14.8	31.21±14.84	28.68±17.44	20.74±13.55	31.09±20.15	27.91±18.3
(range)	(1.67-90.83)	3.33-66.67)	(1.67-70)	(1.67-90.8)	4.17-100	0-65.83	0-100	0-100

Abbreviations: s.d. = standard deviation

Table S3. Summary of all submodels for Finnish and British twins

**Finn Twin Continuous**						**TwinsUK Continuous**					
**Model**	**AIC**	**Comparison Model**	**df**	**Chi-Square**	***p***	**Model**	**AIC**	**Comparison Model**	**df**	**Chi-Square**	***p***
SAT	2953.6	NA	NA	NA	NA	SAT	8447.4	NA	NA	NA	NA
EMO	2949.7	SAT	2	0.04	0.98	EMO	8444.8	SAT	2	1.40	0.50
EMOV	2946.6	SAT	4	0.99	0.91	EMOV	8440.9	SAT	4	1.50	0.83
EMVZ	2943.2	SAT	6	1.58	0.95	EMVZ	8437.4	SAT	6	1.94	0.93
ACE	2945.2	NA	NA	NA	NA	ACE	8433.4	NA	NA	NA	NA
ADE	2945.2	NA	NA	NA	NA	ADE	8433.4	NA	NA	NA	NA
AE	2943.7	ACE	1	0.44	0.51	AE	8431.7	ACE	1	0.26	0.61
	2943.7	ADE	1	0.44	0.51			ADE	1	0.26	0.61
CE	946.4	ACE	1	3.13	0.08	CE	8445.0	ACE	1	13.62	<.001
DE	2943.2	ADE	1	0.00	0.96	DE	8433.4	ADE	1	1.99	0.16
E	2954.2	ACE	2	12.98	<.001	E	8505.0	ACE	2	75.53	<.001
	2954.2	ADE	2	12.98	<.001			ADE	2	75.53	<.001
**Finn Twin Ordinal**						**TwinsUK Ordinal**					
**Model**	**AIC**	**Comparison Model**	**df**	**Chi-Square**	***p***	**Model**	**AIC**	**Comparison Model**	**df**	**Chi-Square**	***p***
SAT	764.3	NA	NA	NA	NA	SAT	2048.8	NA	NA	NA	NA
ETO	759.0	SAT	4	2.66	0.62	ETO	2043.1	SAT	4	2.31	0.68
ETZ	756.4	SAT	6	4.05	0.67	ETZ	2040.1	SAT	6	3.38	0.76
ACE	760.4	NA	NA	NA	NA	ACE	2039.7	NA	NA	NA	NA
ADE	760.4	NA	NA	NA	NA	ADE	2039.7	NA	NA	NA	NA

AE	758.8	ACE	1	0.45	0.50	AE	2037.8	ACE	1	0.09	0.76
		ADE	1	0.45	0.50			ADE	1	0.09	0.76
CE	761.1	ACE	1	2.69	0.10	CE	-	-	-	-	-
DE	758.4	ADE	1	0.00	0.97	DE	-	-	-	-	-
E	767.1	ACE	2	10.67	<.001	E	2067.1	ACE	2	31.38	<.001
		ADE	2	10.67	<.001			ADE	2	31.38	<.001

Note: - indicates model did not converge, AE models chosen in all instances, based on parsimony, AIC, and chi-square tests. EMO tests the equality of means across twin order assumption, EMOV tests the equality of means and variances across twin order assumption, EMVZ tests the equality of means and variances across zygosity assumption, ETO tests the equality of thresholds across twin order assumption, ETV tests the equality of thresholds across zygosity assumption.

Table S4. Summary of overall sex and site differences for continuous and ordinal models

	Chi-Square	df	*p*
	**Continuous Model**		
British sex differences	9.5	4	0.05
Finnish sex differences	3.2	4	0.52
Site differences	76.8	4	<.001
	**Ordinal Model**		
British sex differences	4.4	5	0.49
Finnish sex differences	2.8	5	0.73
Site differences	35.5	5	<.001

Table S5. Summary of site heterogeneity by differences in individual parameters

Parameter Constrained	Chi-Square	df	*p*
**Continuous Model**			
Total Variance	43.1	1	<.001
Heritability	4.2	1	0.04
Age Effect	3.6E-04	1	0.98
Mean Sweet Liking	52.7	1	<.001
**Ordinal Model (Sweet-liking status)**			
Heritability	0.55	1	0.46
Age Effect	0.01	1	0.94
Threshold	0.01	1	0.93
Increment	15.9	1	<.001

Note: Within the ordinal model exploring the heritability of sweet-liking status, the threshold indicates the z-score that an individual needs to move from extreme sweet-liker to moderate sweet-liker, and increment indicates the z-score that an individual needs to move from moderate sweet-liker to sweet-disliker.

Supplement: Armitage et al - Genetic and environmental influences on sweet taste liking and related traits: new insights from twin cohorts

Figure S1A. Heat map of correlations in FinnTwin cohort.

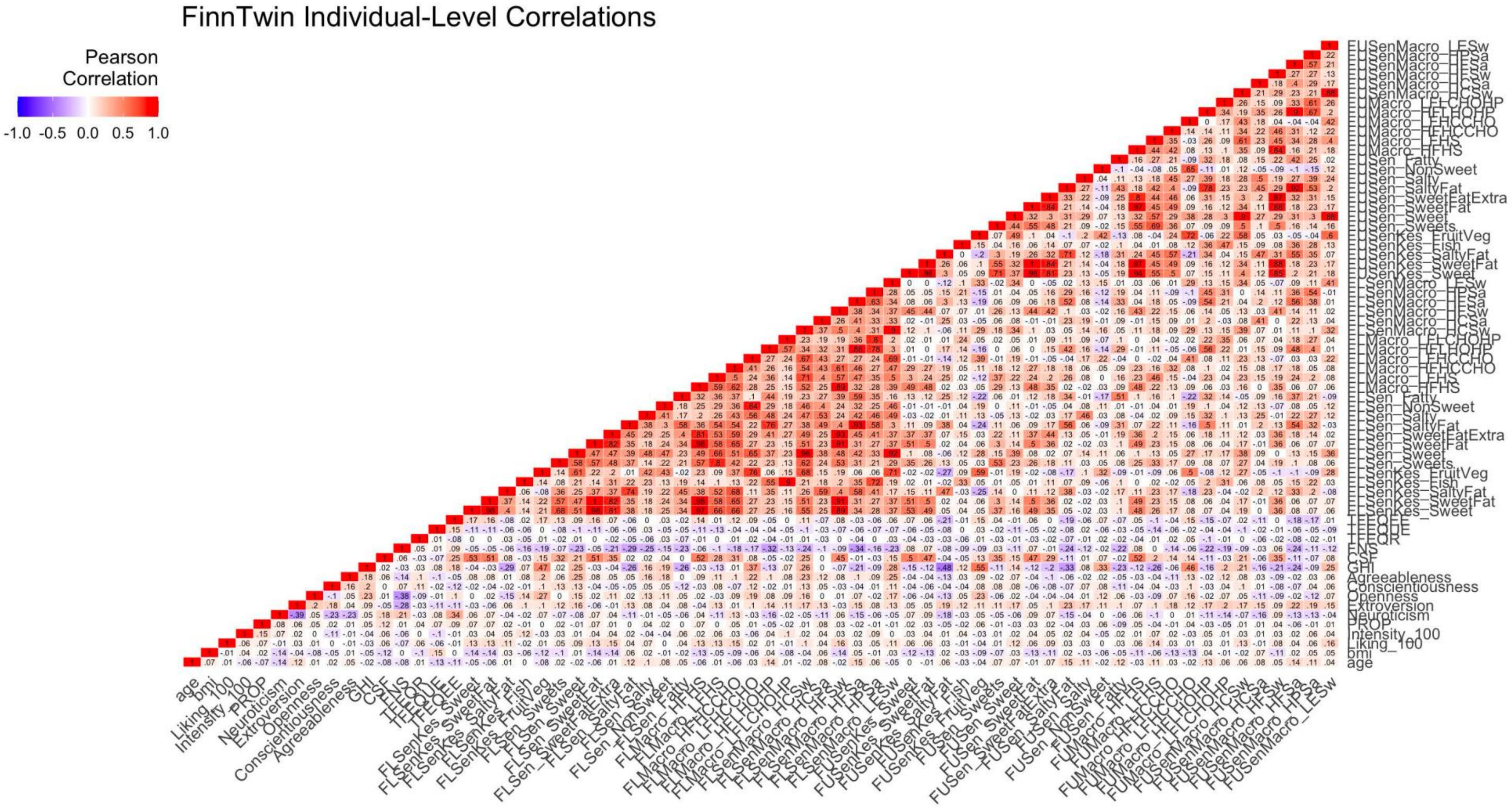

*Abbreviations are provided below **Table S6**.*

Supplement: Armitage et al - Genetic and environmental influences on sweet taste liking and related traits: new insights from twin cohorts

Figure S1B. Heat map of correlations in TwinsUK cohort.

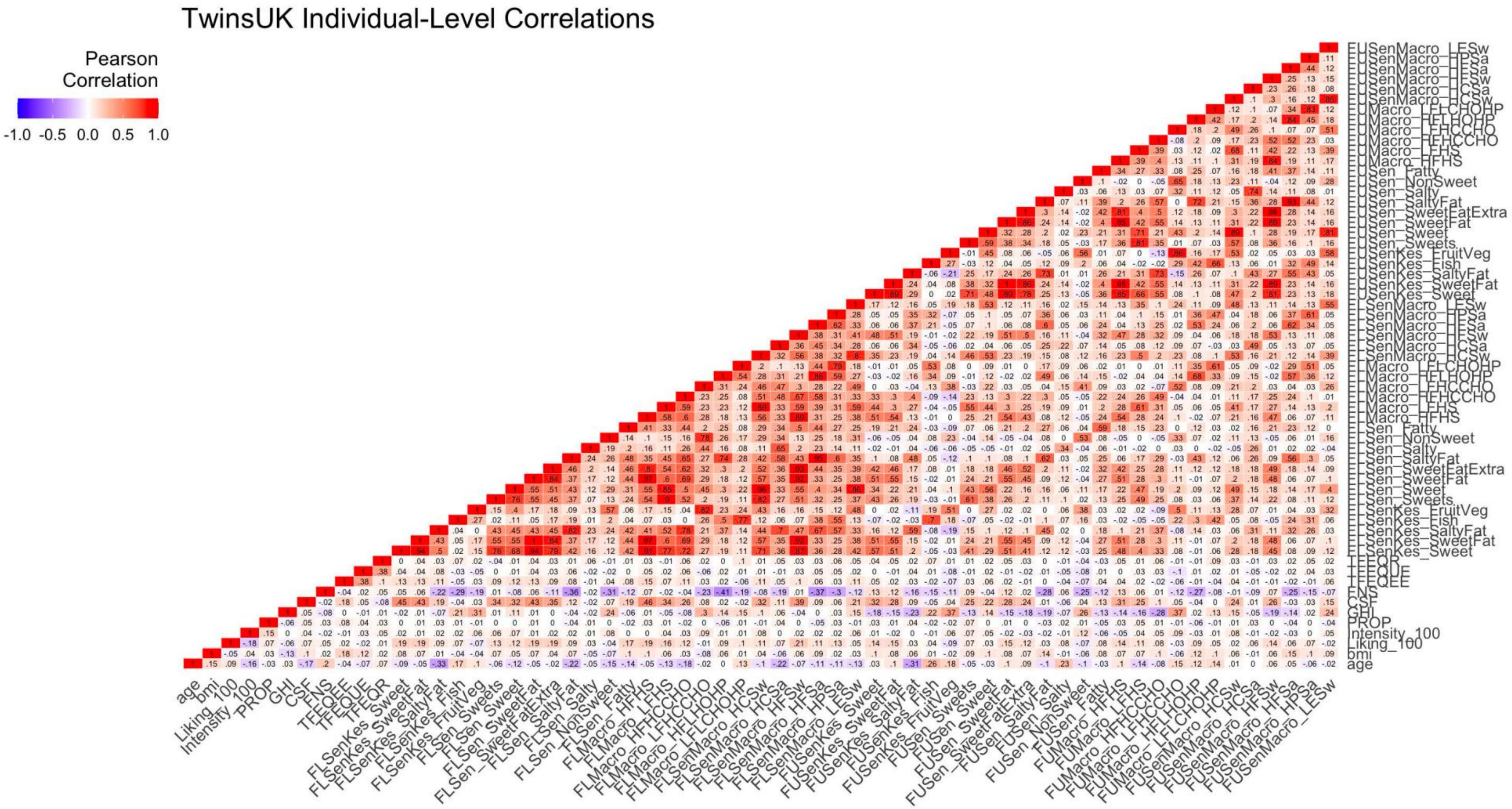

*Abbreviations are provided below **Table S6**.*

Table S6. Summary of *p*-values for correlated variables as shown in Figure S1A and S1B.

Correlated Variable	Category	FinnTwin12 *p*-value	TwinsUK *p*-value
age	Questionnaires, taste tasks and other health measures	0.86	0.0032*
bmi		0.84	0.15
Intensity_100		0.17	0.000000027*
PROP		0.12	0.03*
GHI		0.53	0.07
CSF		0.17	0.03*
FNS		0.15	0.17
TFEQEE		0.76	0.63
TFEQUE		0.19	0.56
TFEQR		0.17	0.89
FLSenKes_Sweet	Food liking, sensory category from Keskitalo's original groupings	0.01*	0.0000000026*
FLSenKes_SweetFat		0.0035*	0.0000000016*
FLSenKes_SaltyFat		0.02*	0.01*
FLSenKes_Fish		0.68	0.03*
FLSenKes_FruitVeg		0.91	0.04*
FLSen_Sweets	Food liking, sensory category	0.28	0.000037*
FLSen_Sweet		0.06	0.00015*
FLSen_SweetFat		0.0035*	0.0000000016*
FLSen_SweetFatExtra		0.00095*	0.0000000052*
FLSen_SaltyFat		0.4	0.0035*
FLSen_Salty		0.12	0.3
FLSen_NonSweet		0.97	0.2
FLSen_Fatty		0.43	0.000000082*
FLMacro_HFHS		0.0038*	0.0000000033*
FLMacro_LFHS		0.0051*	0.00000025*

FLMacro_HFHCCHO	Food liking, macronutrients category	0.0013*	0.0001*
FLMacro_LFHCCHO		0.73	0.65
FLMacro_HFLHOHP		0.78	0.0038*
FLMacro_LFLCHOHP		0.77	0.0017*
FLSenMacro_HCSw	Food liking, sensory-macronutrient category	0.02*	0.00062*
FLSenMacro_HCSa		0.36	0.04*
FLSenMacro_HFSw		0.00047*	0.0000000001*
FLSenMacro_HFSa		0.48	0.0008*
FLSenMacro_HPSa		0.25	0.00009*
FLSenMacro_LESw		0.02	0.1
FUSenKes_Sweet	Food use, sensory category from Keskitalo's original groupings	0.21	0.0000077*
FUSenKes_SweetFat		0.2	0.0000028*
FUSenKes_SaltyFat		0.53	0.36
FUSenKes_Fish		0.77	0.25
FUSenKes_FruitVeg		0.41	0.0034*
FUSen_Sweets	Food use, sensory category	0.81	0.022*
FUSen_Sweet		0.01	0.285
FUSen_SweetFat		0.2	0.0000028*
FUSen_SweetFatExtra		0.06	0.00014*
FUSen_SaltyFat		0.47	0.31
FUSen_Salty		0.56	0.01*
FUSen_NonSweet		0.98	0.04*
FUSen_Fatty		0.48	0.02*
FUMacro_HFHS	Food use, macronutrients category	0.19	0.0000086*
FUMacro_LFHS		0.003*	0.00044*
FUMacro_HFHCCHO		0.5	0.01*
FUMacro_LFHCCHO		0.84	0.4
FUMacro_HFLHOHP		0.51	0.01*

FUMacro_LFLCHOHP		0.87	0.14
FUSenMacro_HCSw	Food use, sensory-macronutrient category	0.01*	0.47
FUSenMacro_HCSa		0.87	0.06
FUSenMacro_HFSw		0.07	0.0000099*
FUSenMacro_HFSa		0.42	0.06
FUSenMacro_HPSa		0.23	0.03*
FUSenMacro_LESw		0.00046*	0.46

Indicates a significant correlation with p set at < 0.05, as shown in* ***Figure S1A* *and* ***S1B****.*

Abbreviations for ***Figure S1A****,* ***Figure S1B*** *and* ***Table S6****.*

Questionnaires, taste tasks and other health measures:

- **bmi**: body mass index
- **Liking_100**: Converted liking scale from 120-mm vertical Labeled Affective Magnitude scale to 100-point rating, used in determining sweet-liking status.
- **Intensity_100**: Converted liking scale from 120-mm vertical Labelled Magnitude scale to 100-point rating.
- **PROP**: 6-n-propylthiouracilfilter paper intensity rating
- **GHI**: Health and Taste Attitude Scales, subscale of General Health Interest
- **CSF**: Health and Taste Attitude Scales, subscale of Craving for Sweet Foods
- **FNS**: The Food Neophobia Scale
- **TFEQ**: Three-Factor Eating Questionnaire (R18)
    - **EE**: emotional eating
    - **UE**: uncontrolled eating
    - **R**: cognitive restraint

Food liking and consumption frequency items: these names are a combination of three parts 1) the questionnaire (FL or FU), 2) the grouping property (sensory: SenKes/Sen; macronutrient: Marco; sensory-macronutrient: SenMacro) and the grouping within listed (listed in sublevels below).

- **FL**: food liking rating
- **FU**: food use rating (i.e., consumption frequency)
- **SenKes**: sensory groupings from Keskitalo's original groupings

  - fatty; fatty-salty; non-sweet; salty; sweet; sweet-fatty; alongside food groupings including fish, fruit and vegetables.
- Sen: sensory groupings from new groupings
  - fatty; fatty-salty; non-sweet; salty; sweet; sweet-fatty; alongside food groupings including fish, fruit and vegetables.
- **Macro**: macronutrient categories
  - HFHS = high fat and high simple sugars;
  - HFHCCHO = high fat and high complex carbohydrates;
  - HFLCHOHP = high fat, low complex carbohydrates and high protein;
  - LFHS = low fat and high simple sugars;
  - LFHCCHO = low fat and high complex carbohydrates;
  - LFLCHOHP = low fat, low complex carbohydrates and high protein
- **SenMarco**: sensory-macronutrient categories:
  - HCS = high carbohydrates and sweet;
  - HCSa = High carbohydrates and salty;
  - HFS = high fat and sweet;
  - HFSa = high fat and high salty;
  - HPSa = high protein and salty

Table S7. Summary of best fitting bivariate results for Finnish and British twins using ordinal model

Correlated Variable	Site	Heritability	Unique Environment	Phenotypic Correlation	Genetic Correlation	Unique Environmental Correlation
**FLMacro_HFHCCHO:** Food liking, macronutrients category of items high fat and high complex carbohydrates	Finland	0.40 [0.23, 0.54]	0.60 [0.46, 0.77]	-0.21 [-0.32, -0.09]	-0.12 [NA, NA]	-0.27 [-0.46, -0.05]
	UK	0.40 [0.30, 0.49]	0.60 [0.51, 0.70]	-0.13 [-0.21, -0.06]	-0.24 [-0.47, -0.02]	-0.06 [-0.20, 0.08]
**FLMacro_HFHS:** Food liking, macronutrients category of items high fat and high simple sugars	Finland	0.53 [0.36, 0.66]	0.47 [0.34, 0.64]	-0.20 [-0.32, -0.08]	-0.14 [NA, NA]	-0.25 [-0.47, -0.02]
	UK	0.50 [0.40, 0.58]	0.50 [0.42, 0.60]	-0.18 [-0.25, -0.10]	-0.38 [NA, -0.18]	0.00 [-0.15, 0.14]
**FLMacro_LFHS:** Food liking, macronutrients category of items low in fat and high simple sugars	Finland	0.33 [0.14, 0.49]	0.67 [0.51, 0.86]	-0.20 [-0.31, -0.08]	-0.03 [-0.10, NA]	-0.29 [-0.50, -0.06]
	UK	0.43 [0.33, 0.52]	0.57 [0.48, 0.67]	-0.17 [-0.24, -0.10]	-0.33 [-0.45, -0.12]	-0.05 [-0.19, 0.09]
**FLSen_SweetFat:** Food liking, sensory category of items sweet and fatty	Finland	0.53 [0.37, 0.66]	0.47 [0.34, 0.63]	-0.20 [-0.31, -0.07]	-0.07 [NA, 0.23]	-0.31 [NA, -0.08]
	UK	0.51 [0.41, 0.59]	0.49 [0.41, 0.59]	-0.18 [-0.26, -0.11]	-0.35 [NA, -0.15]	-0.04 [-0.18, 0.11]
**FLSen_SweetFatExtra:** Food liking, sensory category of sweet and fatty items with additional items	Finland	0.51 [0.34, 0.63]	0.49 [0.37, 0.66]	-0.22 [-0.34, -0.10]	-0.18 [-0.27, 0.05]	-0.26 [-0.46, -0.03]
	UK	0.47 [0.37, 0.56]	0.53 [0.44, 0.63]	-0.17 [-0.25, -0.10]	-0.36 [-0.44, -0.18]	-0.03 [-0.17, 0.12]
**FLSenKes_SaltyFat:** Food liking, sensory category of	Finland	0.36 [0.17, 0.52]	0.64 [0.48, 0.83]	-0.19 [-0.30, -0.07]	-0.29 [NA, NA]	-0.13 [-0.34, 0.09]

salty and fatty items from Keskitalo's original groupings	UK	0.50 [0.41, 0.58]	0.50 [0.42, 0.59]	-0.09 [-0.17, -0.02]	-0.28 [NA, -0.09]	0.07 [-0.07, 0.21]
**FLSenKes_Sweet:** Food liking, sensory category of sweet items from Keskitalo's original groupings	Finland	0.54 [0.38, 0.66]	0.46 [0.34, 0.62]	-0.19 [-0.31, -0.07]	-0.02 [NA, NA]	-0.35 [-0.56, -0.12]
	UK	0.53 [0.43, 0.60]	0.47 [0.40, 0.57]	-0.18 [-0.26, -0.11]	-0.33 [-0.33, -0.13]	-0.05 [-0.20, 0.09]
**FLSenKes_SweetFat:** Food liking, sensory category of sweet and fatty items from Keskitalo's original groupings	Finland	0.53 [0.37, 0.66]	0.47 [0.34, 0.63]	-0.20 [-0.31, -0.07]	-0.07 [NA, NA]	-0.31 [NA, -0.08]
	UK	0.51 [0.41, 0.59]	0.49 [0.41, 0.59]	-0.18 [-0.26, -0.11]	-0.35 [-0.57, -0.15]	-0.04 [-0.18, 0.11]
**FLSenMacro_HCSw:** Food liking, sensory-macronutrient category of high carbohydrates and sweet items	Finland	0.46 [0.30, 0.59]	0.54 [0.41, 0.70]	-0.16 [-0.28, -0.04]	0.03 [-0.32, 0.43]	-0.30 [-0.50, -0.08]
	UK	0.44 [0.33, 0.53]	0.56 [0.47, 0.67]	-0.13 [-0.20, -0.05]	-0.29 [-0.52, -0.07]	0.00 [-0.14, 0.14]
**FLSenMacro_HFSw:** Food liking, sensory-macronutrient category of high fat and sweet items	Finland	0.50 [0.33, 0.63]	0.50 [0.37, 0.67]	-0.25 [-0.37, -0.13]	-0.22 [NA, NA]	-0.28 [NA, -0.05]
	UK	0.50 [0.40, 0.59]	0.50 [0.41, 0.60]	-0.20 [-0.27, -0.12]	-0.36 [NA, -0.15]	-0.06 [-0.21, 0.08]
**FUMacro_LFHS:** Food use, macronutrients category of items low in fat and high simple sugars.	Finland	0.24 [0.07, 0.40]	0.76 [0.6, 0.93]	-0.15 [-0.27, -0.03]	-0.24 [-0.41, NA]	-0.12 [-0.32, 0.10]
	UK	0.41 [0.30, 0.50]	0.59 [0.50, 0.70]	-0.14 [-0.21, -0.06]	-0.18 [-0.40, 0.05]	-0.11 [-0.24, 0.04]

Note: Correlations are largely negative because in the ordinal model, sweet-liking status is ordered from extreme sweet-liker to moderate sweet-liker and, lastly, sweet disliker. Negative correlations here indicate that less sweet disliking (i.e., greater sweet liking) is associated with greater liking or use of various food types. NA indicates that the confidence interval bound did not converge.

Table S8. Summary of characteristics by age group for Finnish and British twins

	**Finnish Twins**	**British Twins**						
	Twenties	Teens	Twenties	Thirties	Forties	Fifties	Sixties	Seventy+
**BMI**								
mean±s.d.	23.45±3.88	24.09±3.25	23.95±3.36	24.64±4.35	25.99±5.47	26.59±4.72	26.68±4.3	27.21±4.73
(range)	(17.21-42.91)	19.6-29.57	18.95-32.68	17.8-39.55	18.34-48.12	17.51-42.17	16.9-41.42	17.75-48.57
**Liking**								
mean±s.d.	60.29±14.8	63.44±21.49	50.62±17.6	51.75±23.02	48±20.74	51.98±19.05	54.16±19.49	57.45±17.17
(range)	13.33-95.83	20.83-81.67	17.5-87.5	0-87.5	0-90.83	0-89.17	0-91.67	9.17-95
**Intensity**								
mean±s.d.	31.21±14.84	38.12±16.95	36.85±15.05	33.43±21.98	29.64±18.4	27.45±18.44	26.4±16.67	23.41±18.11
(range)	(1.67-90.8)	17.5-62.5	6.67-55.83	0-102.5	0.83-102.5	1.67-102.5	1.67-102.5	0-102.5

Abbreviations: s.d., standard deviation

Table S9. Summary of characteristics by sex for Finnish and British twins

	**Finnish Twins**		**British Twins**	
	Male	Females	Male	Females
**BMI**				
mean±s.d.	24.26±3.96	22.92±3.75	26.83±3.49	26.27±4.83
(range)	(17.29-42.91)	(17.21-38.92)	18.34-26.03	16.9-48.57
**Liking**				
mean±s.d.	61.34±14.33	59.6±15.08	57.29±17.19	52.07±19.91
(range)	(21.67-88.33)	(13.33-95.83)	9.17-87.5	0-95
**Intensity**				
mean±s.d.	31.63±14.3	30.93±15.21	25.54±14.63	28.19±18.68
(range)	(3.33-84.17)	(1.67-90.83)	4.17-76.76	0-100

Abbreviations: s.d., standard deviation
